# Co‐design workshops to inform an at‐home rehabilitation intervention for people with dementia: a qualitative analysis

**DOI:** 10.1002/alz70858_105897

**Published:** 2025-12-26

**Authors:** Clare Stephenson, Kasia Bail, Diane Gibson, Sophia Hadjimichael, Stephen Isbel, Rachael Mitterfellner, Michelle Bennett, Nathan M D'Cunha

**Affiliations:** ^1^ University of Canberra, Bruce, ACT, Australia; ^2^ Canberra Health Services, Canberra, ACT, Australia; ^3^ Canberra Health Services, Bruce, ACT, Australia

## Abstract

**Background:**

At‐home rehabilitation programs for people with dementia require essential components that are both person‐centred and goal‐directed. While several interventions have been evaluated in Australia, they often require significant resources and time to deliver. This research investigates end‐user priorities for developing a cost‐effective and accessible home‐based dementia rehabilitation program, for people unable to participate in group settings in the Australian Capital Territory.

**Method:**

A co‐design working group was established to ensure the views of all stakeholders contributed to the intervention design, which has funding allocated for six home visits per participant. An occupational therapist and physiotherapist will design and monitor the intervention which will be delivered by an allied health assistant. Four two‐hour co‐design workshops were held over a period of six months. Sixteen experts were recruited, comprising three dyads, and ten healthcare professionals with a minimum of three years cumulative experience working with people with dementia. Workshops were recorded and transcribed, and iterative thematic analysis was applied.

**Result:**

Four overarching themes and seven subthemes were identified. Themes: 1) Awareness, acceptance and access; 2) Person‐centred care: Understanding unique needs, preferences, and goals, with two subthemes: a) Respect and dignity, b) Flexibility and adaptability; 3) Empowerment through community engagement & continuous support, with three subthemes: a) Building confidence & self‐efficacy. b) A gateway to engaging with other services c) Preventing information overload & overwhelm; and 4) Multidisciplinary coordinated care, with two subthemes: a) creating a raft and b) ensuring robust handover processes.

**Conclusion:**

Engaging relevant stakeholders in the co‐design process helped to identify key components deemed feasible and valuable for an at‐home intervention for people with dementia. Findings demonstrate confidence from participants that the program would be feasible and valuable to people with dementia living at home. Assessing the feasibility and effectiveness of the at‐home rehabilitation program is necessary to determine whether it should be implemented in the public health system in the Australian Capital Territory.

